# Supplementary material for: Gut Microbiota-Derived PGF2α Fights against Radiation-Induced Lung Toxicity through the MAPK/NF-κB Pathway
Source: Antioxidants (Basel). 2021 Dec 28;11(1):65. doi: 10.3390/antiox11010065 (PMC8773112; doi:10.3390/antiox11010065)
Supplement: Supplementary file 1 [file antioxidants-11-00065-s001.zip › antioxidants-1507344-supplementary.pdf]

Supplementary Material

1. Supplementary figures and figure legends

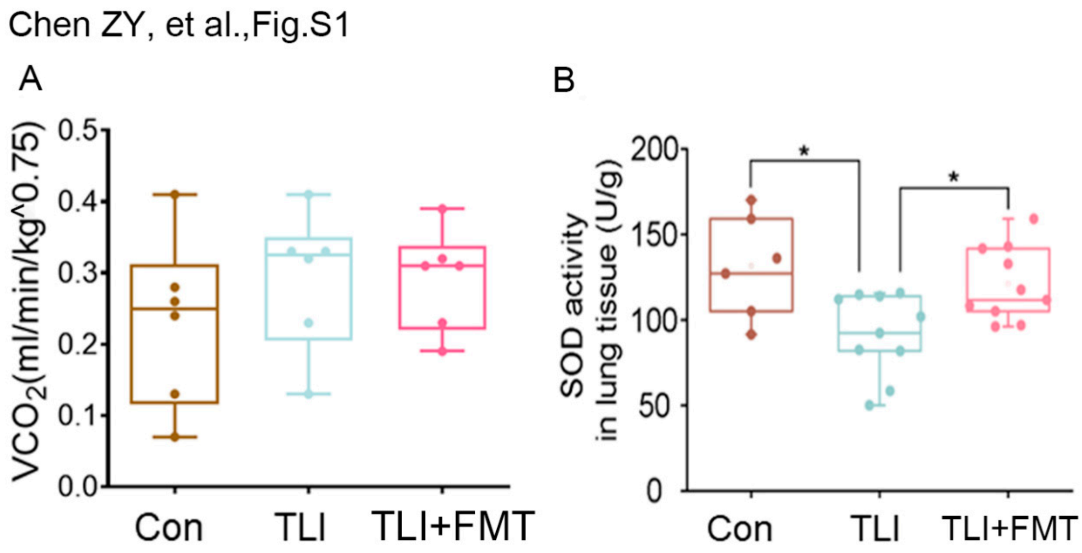

Figure S1 Faecal microbiota transplantation fights against radiation pneumonia in mice **(A)** VCO_2 excretion of mice in each group in 24 h (n=6); **(B)** The SOD expression in lung tissues of each group (Con: n=6, TLI and FMT: n=10). ($^{*}p < 0.05$, $^{**}p < 0.01$ and $^{***}p<0.001$; Student's t test)

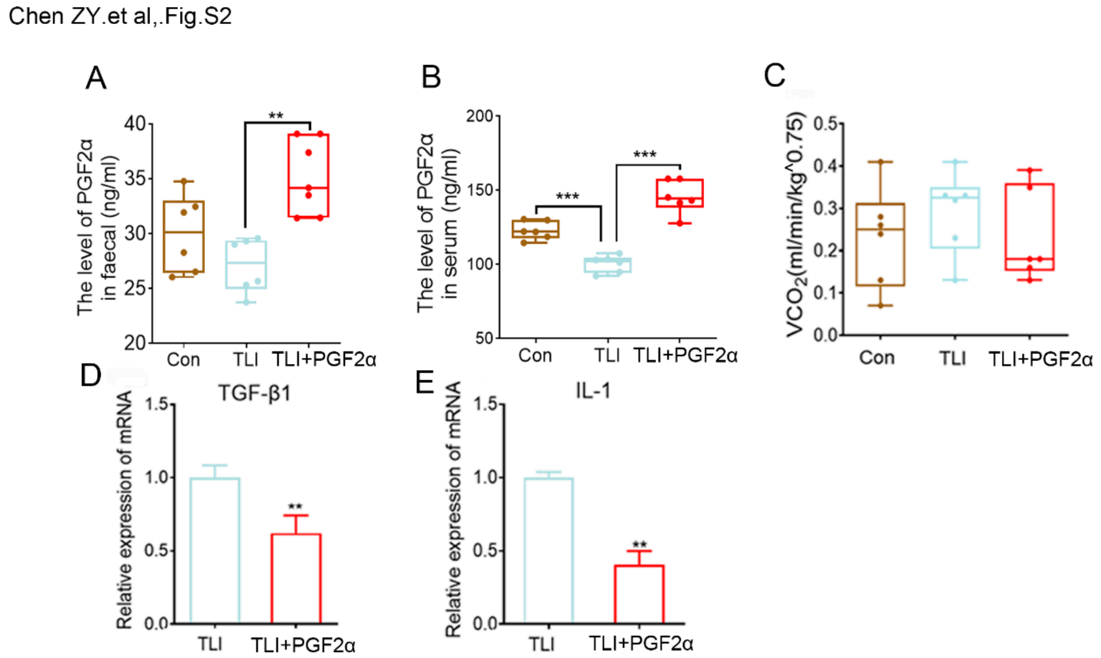

Figure S2 Metabolite PGF2α improves radiation pneumonitis in mice. **(A)** The level of PGF2α in faecal (ELISA, n=8); **(B)** The level of PGF2α in serum (ELISA, n=8). **(C)**

VCO2 of mice in each group in 24 h (n=6). (**D, E**) The expression of TGF-β1 and IL-1 mRNA. ($^{*}p < 0.05$, $^{**}p < 0.01$; $^{***}p < 0.005$; Student's *t* test; PGF2α:16 μg/ml, 200 μl / mouse / day)

Chen ZY, et al.,Fig.S3

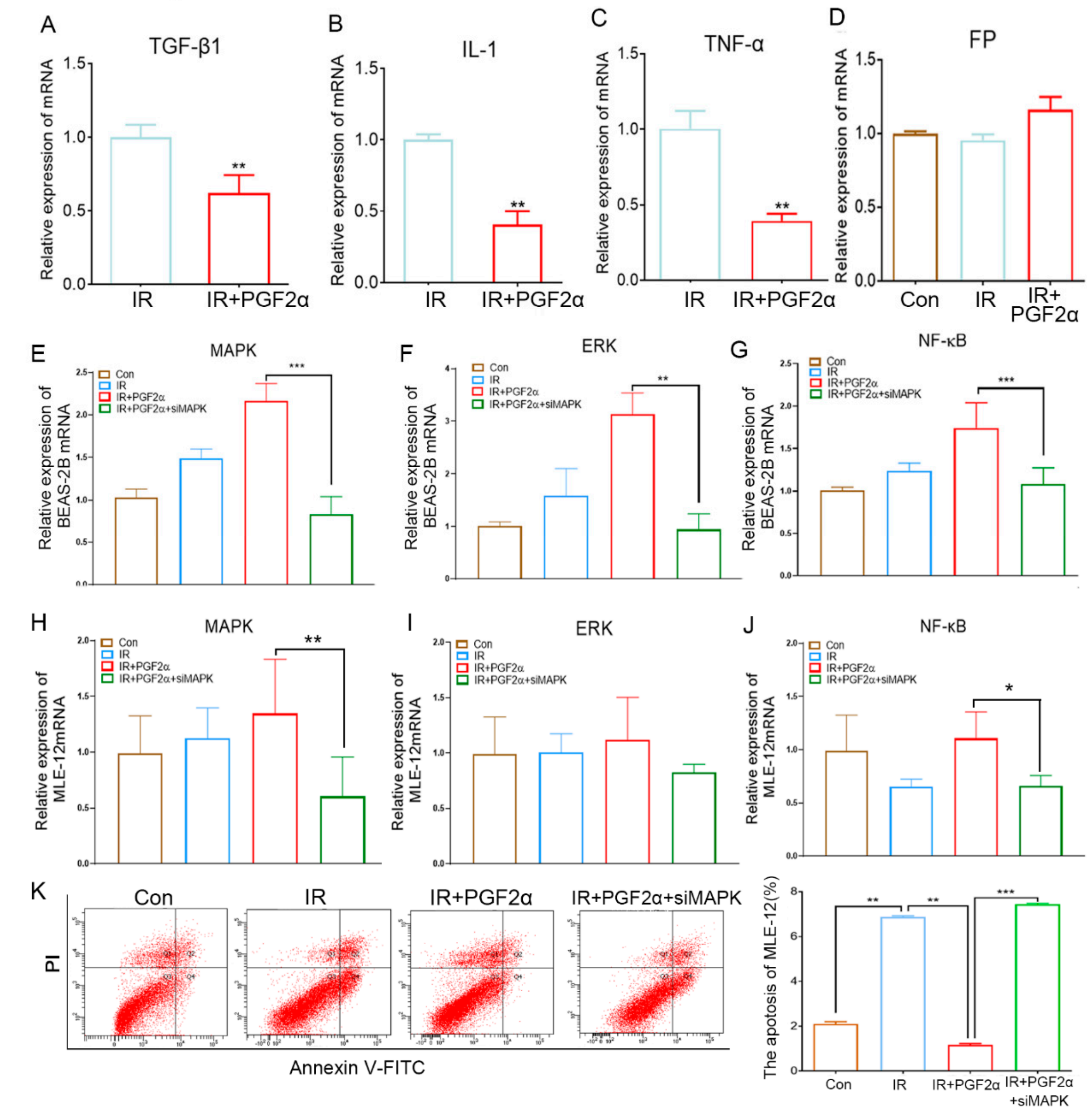

Figure S3 Data on changes in RNA level expression of related genes and apoptosis after PGF2α and si-MAPK treatment **(A-D)** The TGF-β1, IL-1, TNF-α and FP expression changes at the mRNA level. **(E-G)** The expression levels of MAPK, ERK and NF-κB in irradiated BEAS-2B cells were assessed by qRT-PCR. **(H-J)** The expression levels of MAPK, ERK and NF-κB in irradiated MLE-12 cells were assessed by qRT-PCR. **(K)** The apoptosis of irradiated MLE-12 cells was analyzed by flow cytometry. ($^{*}p < 0.05$, $^{**}p < 0.01$; Student's *t* test; n=3)

2. Supplementary table 1

List of primers used in this paper.

Gene	Primer	Sequence (5′-3′)
**Primers for PCR**		
GAPDH	forward	TGTTTCCTCGTCCCGTAGA
	reverse	CAATCTCCACTTTGCCACTG
TNFa	forward	TTCTCATTCCTGCTTGTGGCA
	reverse	ACTTGGTGGTTTGCTACGACG
IL-1	forward	TTGAAGAAGAGCCCATCCTC
	reverse	CAGCTCATATGGGTCCGAC
NF-KB	forward	GAAGCACGAATGACAGAGGC
	reverse	GCTTGGCGGATTAGCTCTTTT
TGF-beta1	forward	GGCCAGATCCTGTCCAAGC
	reverse	GTGGGTTTCCACCATTAGCAC
Pi3k	forward	AGAGCACTTGGTAATCGGAGG
	reverse	CTTCCCCGGCAGTATGCTTC
AKT	forward	AGCGACGTGGCTATTGTGAAG
	reverse	GCCATCATTCTTGAGGAGGAAGT
FP	forward	GAGCGGTGTATTGGAGTCACA
	reverse	GTCCTCGACGCCTGAATTTTA
**Primers for sequencing**		
515F		GTGCCAGCMGCCGCGGTAA
806R		GGACTACHVGGGTWTCTAAT

3. The whole uncropped images of the original western blots

Western blotting analysis using the following antibodies: caspase6(1:1000, Cat #ab52951; Abcam, USA), JNK (1:500, Cat#ab31419, Abcam) phospho-ERK (1:1000, Cat#ab201015; Abcam), MAPK (p38) (1:1000, Cat #ab185145; Abcam), phospho-AKT (1:1000, Cat #ab38449; Abcam), phospho-PI3Kinase (1:1000, Cat #ab182651; Abcam), NF-κB (1:500, #SC-8008; SANTA CRUZ),GAPDH(1:5000, #60004;Proteintech, USA).

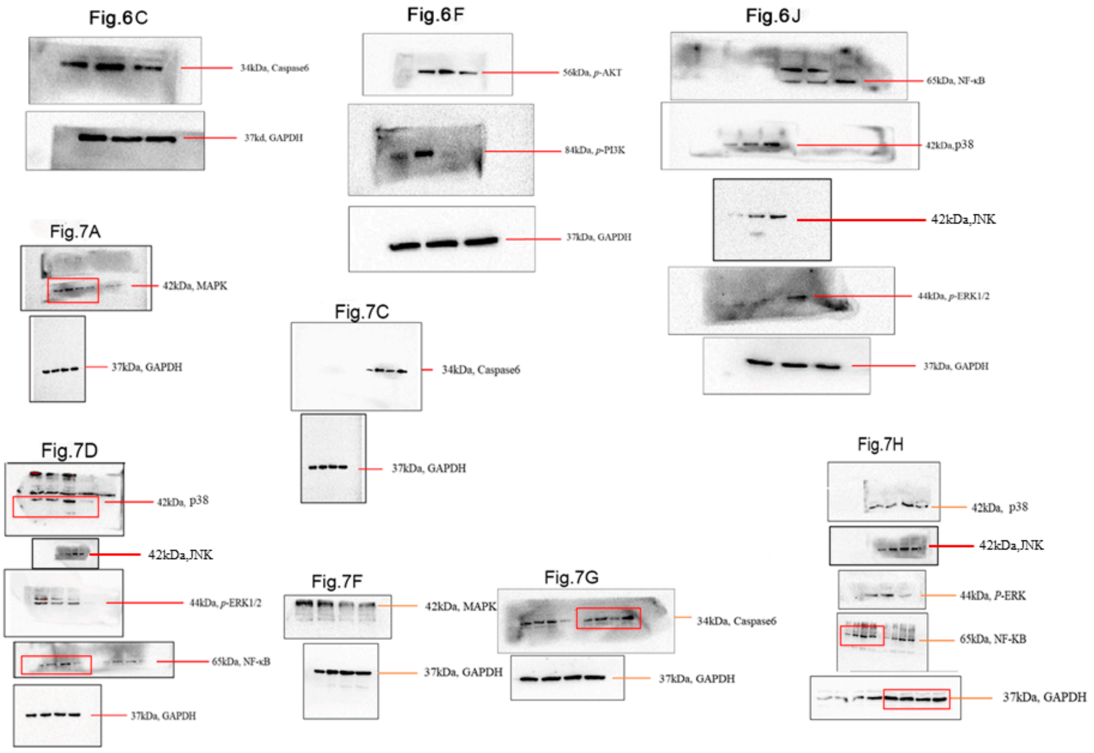

Fig.6C
34kDa, Caspase6
37kd, GAPDH
Fig.6F
56kDa, p-AKT
84kDa, p-PI3K
37kDa, GAPDH
Fig.6J
65kDa, NF-κB
42kDa,p38
42kDa,JNK
44kDa, p-ERK1/2
37kDa, GAPDH
Fig.7A
42kDa, MAPK
37kDa, GAPDH
Fig.7C
34kDa, Caspase6
37kDa, GAPDH
Fig.7D
42kDa, p38
42kDa,JNK
44kDa, p-ERK1/2
65kDa, NF-κB
37kDa, GAPDH
Fig.7F
42kDa, MAPK
37kDa, GAPDH
Fig.7G
34kDa, Caspase6
37kDa, GAPDH
Fig.7H
42kDa, p38
42kDa,JNK
44kDa, P-ERK
65kDa, NF-KB
37kDa, GAPDH
